# On the Effects of Conium Maculatum.—Spurious Medicines

**Published:** 1846-03-01

**Authors:** 


*On the Effects of Conium Maculatum—Spurious Medicines.*—In the first No. of our Journal we adduced a statement contained in a report by Dr. Woodward, of Worcester, Mass., that the Conium Maculatum to produce any effects, must be given in doses of 30 or 40 grains, or more. In our 5th No. we quoted a series of experiments by Pliny Earl, M. D., which tended to confirm this statement. The dose, as laid down in the Dispensatories, is from 3 to 4 grains. In commenting on the above communications we intimated that the explanation of the discrepancy consisted in the fact that the extracts employed by these gentlemen were improperly prepared, or had become nearly inert. It would surely be surprising, if, in the long experience of the profession with this drug, it should not have been before discovered that the doses usually administered are entirely without effect. The explanation which we suggested is sustained by some experiments of Dr. Fountain, of Somers, Westchester Co., N. Y., which are contained in the January No. of the Am. Jour. Med. Sciences. Dr. F. prepared the extract with which his experiments were made himself, and employed it while in a fresh state. The effects of twelve grains which he administered to himself were very decided, causing dimness of vision, ocular spectra, numbness and stiffness in the muscles of the upper extremities, and incomplete paralysis of the lower extremities. The pulse was soft and full, but not accelerated. These effects continued more or less through the day. Afterwards, four grains given to a patient, produced unpleasant effects. The reader is referred to the article alluded to for more full details. The writer deduces from his observations the importance of using well prepared and fresh extracts. He advises that practitioners prepare it for themselves, as the plant grows luxuriantly through the country, or, at least, that they see to it that Druggists provide themselves with a veritable article.

This subject suggests that of spurious medicines generally. We have reason to believe that a large proportion of the drugs with which practitioners are supplied, and which are dispensed by apothecaries, are deficient in purity, either from imperfect preparation, or adulteration. The editor of the New-York Journal, in the number for September last, announced his intention of giving an *expose* of some startling facts in his possession on this subject. It has not, however, as yet appeared; but we trust he has not relinquished his intention. The profession generally are doubtless, not aware of the extent to which this imposition is carried on, and it is highly important that the facts should be known both by the profession and the public. The responsibility lies both with druggists and physicians. The former may not be competent to determine the purity of the articles which they vend; and the latter seldom take pains to do so. We refer now particularly to those practitioners who dispense medicines to their patients, as is generally the case except in large cities. We are sorry to say that Physicians not only omit the duty of close examination of the quality of the medicines they use, but sometimes obtain those of an inferior quality, knowingly, because they can be purchased at a cheaper rate. We have been told by Druggists that they are obliged to keep articles of an inferior quality to suit this class of customers. This abominable practice cannot be reprehended in too severe terms. What ideas can such persons have of the practice of medicine, and their obligations to their patients! For the honor of the profession we trust such instances are not numerous; but we are compelled to believe that habitual inattention to the selection of medicines is quite common.

The importance of precise and uniform properties in medicinal substances is too obvious to require illustration. Established principles of therapeutics, and the practical precepts of every member of the profession, as a matter of course, are based upon them. In proportion to their variableness, scientific observations, and individual experience involve incongruities and errors. Doubtless it is to this source that much of the discrepancy of facts appertaining to different medical records and opinions is to be attributed. The uncertainty which attaches to this point always imposes the necessity of a conditional acknowledgement of deductions from the trial of remedies, if adequate testimony of the purity of the medicines be not at the same time given. These considerations, the force of which is sufficiently apparent, assuredly make the subject one of great moment. Our contemporary of the New-York Journal is entitled to much credit for broaching it, and if he will prosecute his intention as announced, he will render a most valuable service to the profession, to science and humanity.

The practical question comes up in connection with these remarks, how can the evils referred to be remedied and effectually prevented? The observance of greater caution on the part of conscientious apothecaries and physicians will do much, but this is not all, as it seems to us, that the subject demands. The truth is, it is a subject in which Legislation ought to interfere, and protect the safety and interest of the public, by establishing properly qualified inspectors of medicines, and imposing high penalties for vending or dispensing drugs which have not passed the ordeal of a thorough examination. The public see the propriety of making necessary articles of food, and even some luxuries, subject to inspection before sale; but concerning medicines, with regard to which most persons can exercise no discrimination whatever, the necessity of some legal precautions has never been recognised. Are not the dangers to which the public is exposed from inert and adulterated drugs quite as great as from poor flour, pork, fish or tobacco? It is not probable, however, that an effort to induce legislators to act in this matter for the public weal would be successful. We should perhaps be accused of some sinister motives—some scheme of monopoly. It remains then to enquire if the Profession cannot do something, without the aid of Legislation, to correct the evils which now exists. It appears to us that if this matter were to be taken up by the national convention, or by the permanent association, which, it is to be hoped, will grow out of this convention, commissioners might be appointed in our larger cities, under whose supervision medicines should pass, and it should be recommended to the profession generally to repudiate all drugs which had not received their sanction. This plan would promote the interests both of practitioners and respectable druggists, and both would, we conceive, readily fall in with it. The detailed provisions necessary to carry this improvement into effect, we will not now consider. Perhaps there are difficulties in the way of its successful operation which do not occur to us. We give the idea as it has suggested itself without having bestowed upon this branch of the subject lengthened reflection. Possibly legislation might be more amenable to the public welfare than we anticipate, but here a difficulty would arise in a want of co-operation or uniformity in different states. Since none can doubt the great importance of the object, whatever may be the way in which it can be most readily and effectually obtained, we trust some good may follow the effort to direct the attention of the profession toward it.

